# Lipid profile-based prognostic nomograms for obstructive colorectal cancer: multi-cohort development and validation

**DOI:** 10.3389/fmed.2026.1910496

**Published:** 2026-07-27

**Authors:** Hanwenchen Wang, Falong Zou, Denglong Cheng, Jun Wang, Mian Chen, Junnan Gu, Fuwei Mao, Kun Dai, Ke Wu, Hongli Liu, Xiu Nie, Li Liu, Jianguo Shi, Shenghe Deng, Kailin Cai

**Affiliations:** 1 Department of Gastrointestinal Surgery, Union Hospital, Tongji Medical College, Huazhong University of Science and Technology, Wuhan, Hubei, China; 2 Department of Thoracic Surgery, Union Hospital, Tongji Medical College, Huazhong University of Science and Technology, Wuhan, Hubei, China; 3 School of Nursing, Wuhan University, Wuhan, China; 4 Cancer Center, Union Hospital, Tongji Medical College, Huazhong University of Science and Technology, Wuhan, Hubei, China; 5 Department of Pathology, Union Hospital, Tongji Medical College, Huazhong University of Science and Technology, Wuhan, Hubei, China; 6 Department of Epidemiology and Biostatistics, School of Public Health, Tongji Medical College, Huazhong University of Science and Technology, Wuhan, China; 7 Department of Gastrointestinal Surgery, The Central Hospital of Wuhan, Tongji Medical College, Huazhong University of Science and Technology, Wuhan, China; 8 Department of General Surgery, Union Hospital, Tongji Medical College, Huazhong University of Science and Technology, Wuhan, Hubei, China; 9 Center for Liver Transplantation, Union Hospital, Tongji Medical College, Huazhong University of Science and Technology, Wuhan, Hubei, China

**Keywords:** colorectal cancer, disease-free survival (DFS), lipid profile, nomogram, obstruction, OS

## Abstract

Patients with obstructive colorectal cancer (OCRC) have poor prognosis, and conventional prognostic tools such as TNM staging alone are insufficient for accurate assessment; thus, this study aimed to develop and validate nomograms for predicting overall survival (OS) and disease-free survival (DFS) in OCRC patients. A total of 1,650 eligible colorectal cancer patients were enrolled, including 1,520 from Center A (randomly split 6:4 into training and internal validation cohorts) and 130 from Center B as the external validation cohort; clinicopathological and lipid profile data were collected retrospectively, independent prognostic factors were identified via univariate and multivariate Cox regression, and OS/DFS nomograms were constructed and evaluated using ROC curves, C-index, calibration curves, and DCA. The OS nomogram included 8 factors with 1-, 3-, 5-year AUCs of 0.761–0.793 (training), 0.759–0.788 (internal validation), and 0.723–0.856 (external validation), while the DFS nomogram included 6 factors with corresponding AUCs of 0.777–0.822, 0.750–0.841, and 0.775–0.820, and all evaluations confirmed excellent model performance. In conclusion, two nomograms incorporating lipid and clinicopathological factors were developed and validated, which effectively predict OS and DFS in OCRC patients with good discrimination, calibration, and clinical utility, providing new tools for individualized risk prediction.

## 1 Introduction

Colorectal cancer (CRC) is the third most common malignancy and the second leading cause of cancer-related death worldwide. Management of its complications remains a critical focus in clinical oncology (1, 2). Among these, mechanical obstruction is the most prevalent, with approximately 8%–29% of CRC patients presenting with acute intestinal obstruction as their first clinical manifestation, forming a distinct subtype known as obstructive colorectal cancer (OCRC) (3, 4). OCRC is defined by the presence of clinical symptoms of bowel obstruction, such as abdominal pain, bloating, vomiting, and inability to pass gas or stool, in combination with any one of the following positive diagnostic findings: abdominal X-ray showing gas-fluid levels, CT imaging demonstrating proximal bowel dilatation and distal bowel collapse at the site of obstruction, or the inability to pass the endoscope through the obstructed segment as confirmed by colonoscopy (5–8). Additionally, the incidence of OCRC has been rising annually, with a notable increase in younger patients, presenting a significant challenge (9).

This subtype exhibits significantly different prognostic characteristics compared to non-obstructive CRC. Multiple studies have shown that OCRC patients have poorer short-term outcomes and worse 5-year OS and DFS than non-obstructive CRC patients. Specifically, OCRC patients exhibit higher 90-day postoperative mortality (10.0% vs. 3.0%), lower 5-year OS (32.0%–67.7% vs. 60.0%–78.0%), and lower 5-year DFS (53.9% vs. 67.0%) (10–12). All studies consistently identify obstruction as an independent risk factor for poor prognosis. These findings highlight the critical need for early identification and the development of personalized management strategies for OCRC, as it presents unique challenges to both short-term and long-term patient prognosis.

The controversy surrounding OCRC treatment strategies highlights the limitations of existing prognostic assessment tools. Emergency resection (ER) can rapidly relieve obstruction but is associated with high postoperative complication and mortality rates, often necessitating a permanent stoma, which severely impacts the patient’s quality of life (13, 14). Bridge-to-surgery (BTS), utilizing self-expanding metal stents (SEMS) and decompression stomas (DS), improves short-term outcomes but does not significantly enhance long-term survival (15, 16). An innovative strategy combining SEMS and neoadjuvant chemotherapy (SEMS-NAC) has shown good short-term clinical benefits in a prospective study (17), but safety concerns persist. The European Society of Gastrointestinal Endoscopy (ESGE) guidelines support the use of chemotherapy after stent placement (18), whereas the Japanese Society for Cancer of the Colon and Rectum (JSCCR) guidelines suggest that chemotherapy may increase the risk of stent perforation (19, 20). These controversies reflect the central dilemma in OCRC treatment: the absence of precise prognostic assessment tools to guide individualized treatment decisions (21). Current prognostic tools mainly focus on CRC as a broader group, utilizing common tumor-node-metastasis (TNM) staging and relying on conventional tumor markers such as carcinoembryonic antigen (CEA), carbohydrate antigen 19-9 (CA19-9), and systemic indicators like the prognostic nutritional index (PNI), neutrophil-lymphocyte ratio (NLR), and lymphocyte-monocyte ratio (LMR) (22–25). However, these tools have significant limitations when applied to OCRC (10, 26). Studies have shown that even patients with the same TNM stage in OCRC can experience significantly different survival outcomes. This heterogeneity arises from the failure of existing models to account for obstruction-specific pathological mechanisms, such as extracellular matrix remodeling and metabolic abnormalities (27–29).

Notably, lipid metabolism abnormalities have been shown to be closely linked to invasion, metastasis, and poor prognosis in various solid tumors (30, 31). However, their role in OCRC has not been sufficiently explored (32, 33). Our team recently discovered that the abnormal accumulation of palmitic acid within OCRC tumors activates the NF-κB pathway in tumor cells, inducing the transformation of matrix cancer-associated fibroblasts (mCAFs). This transformation promotes extracellular matrix remodeling and contributes to OCRC progression (34). To our knowledge, this is the first study to reveal a direct connection between lipid metabolism abnormalities and OCRC progression, providing a theoretical foundation for the development of OCRC-specific, precise prognostic prediction tools. Palmitic acid, a saturated fatty acid, is closely associated with lipid metabolism. It influences liver lipid accumulation, leading to increased levels of TG and CHOL (35, 36). Clinical study has also shown that a palmitic acid-rich diet significantly raises LDL-C levels (37). Building on this, our study is the first to incorporate lipid metabolism abnormalities into prognostic models for OCRC. By emphasizing OCRC-specific metabolic factors, these models offer a distinct approach compared to previous models that primarily focused on tumor markers or inflammatory indices (22–25).

Addressing this research gap and leveraging mechanistic breakthroughs, our study integrates clinical lipid profiles, including TG, LDL-C, HDL-C, and CHOL, with atherosclerosis-related indicators such as AAS and CAS. These are combined with conventional clinical and pathological features to develop more accurate and efficient nomograms for predicting OS and DFS in OCRC patients. We hypothesize that integrating lipid profile data and atherosclerosis-related indicators will significantly enhance the prognostic accuracy for OCRC patients, ultimately improving risk assessment and optimizing treatment strategies. This provides evidence-based support for risk assessment and treatment strategy optimization, while also clarifying the relationship between lipid metabolism features and clinical outcomes to promote personalized precision medicine for OCRC patients.

## 2 Materials and methods

### 2.1 Study design and patients

**FIGURE 1:**
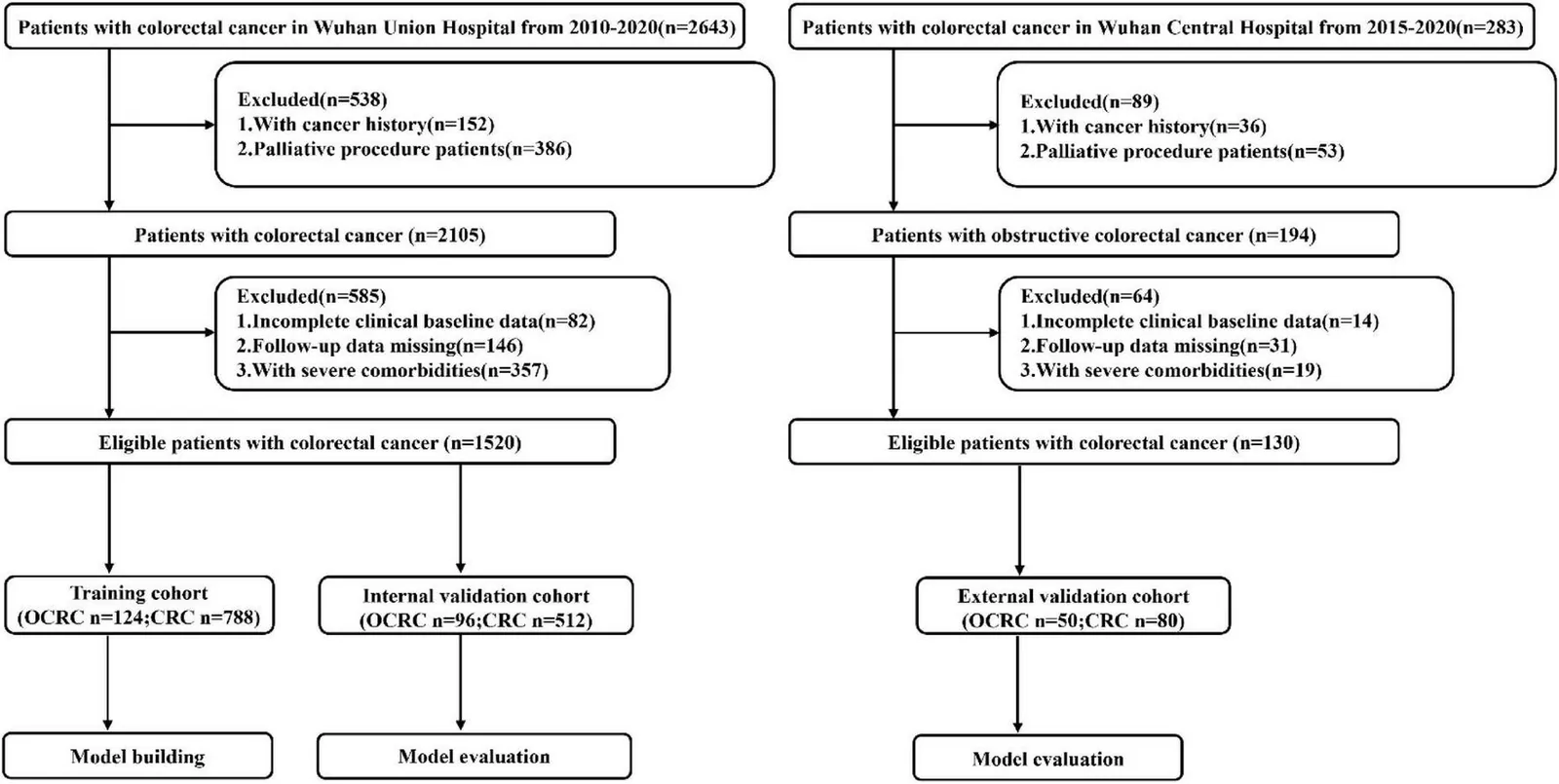
Flowchart of the patient selection process for this study.

This study included 1,650 CRC patients from partner institution A (a tertiary medical center; *n* = 1,520) and partner institution B (another tertiary medical center; *n* = 130). At center A (surgeries performed between 2010 and 2020), 220 patients (14.5%) presented with obstruction and the full center-A cohort was randomly split 6:4 into a training set [*n* = 912; OCRC = 124 (13.6%)] and an internal validation set [*n* = 608; OCRC = 96 (15.8%)]. At center B (surgeries performed between 2015 and 2020), 50 of 130 patients (38.5%) had obstructive disease, constituting the external validation cohort (OCRC = 50). OCRC was defined by the presence of clinical symptoms of bowel obstruction, such as abdominal pain, bloating, vomiting, and inability to pass gas or stool, in combination with any one of the following positive diagnostic findings: abdominal X-ray showing gas-fluid levels, CT imaging demonstrating proximal bowel dilatation and distal bowel collapse at the site of obstruction, or the inability to pass the endoscope through the obstructed segment as confirmed by colonoscopy, per ACPGBI/ESGE/FSID/EAST (5–8). These OCRC cases formed the population for nomogram development (training) and validation (internal/external). The inclusion criteria were as follows: (1) pathologically confirmed CRC; (2) radical surgical resection; and (3) availability of complete clinical, pathological, and follow-up data. Exclusion criteria were as follows: (1) history of other malignancies; (2) palliative treatment instead of radical surgery; (3) incomplete baseline clinical data; (4) missing follow-up data; and (5) severe comorbidities, defined as conditions such as ASA IV patients or those with a limited life expectancy from other causes (e.g., advanced heart failure, end-stage renal disease), were excluded. The complete patient selection process is shown in Figure 1. The study design and reporting adhere to the STROCSS criteria (38). All procedures fully adhered to the tenets of the Declaration of Helsinki. The requirement for informed consent was waived owing to the retrospective nature of the study. The study protocol was reviewed and approved by the Ethics Committee of institution A (Approval No. 2018-S377) and institution B (Approval No. 2021-31).

### 2.2 Data collection

Demographic and clinicopathological data were collected, including age, sex, body mass index (BMI), tumor differentiation, TNM stage, T stage, N stage, M stage, tumor location, tumor size, circumferential resection margin (CRM), obstruction status, vascular lymphatic invasion (VI), perineural invasion (PNI nerve), triglycerides (TG), total cholesterol (CHOL), low-density lipoprotein cholesterol (LDL-C), and high-density lipoprotein cholesterol (HDL-C). All clinicopathological factors were classified according to the eighth edition of the American Joint Committee on Cancer (AJCC) staging system. BMI and TG, CHOL, LDL-C, and HDL-C levels were stratified according to the Chinese Obesity Diagnosis and Treatment Guidelines and the Chinese Lipid Management Guidelines, as outlined in Supplementary Table 1. For the purposes of Cox regression analysis and nomogram construction, BMI and lipid levels were used as categorical variables based on these clinical guidelines (e.g., TG ≥ 1.7 mmol/L as “High”). This approach aligns with clinical practice for risk stratification and improves the interpretability of the model. The study endpoints were OS and DFS. OS was defined as the time from radical surgery to death from any cause or the end of follow-up, whichever occurred first. DFS was defined as the time from radical surgery to the first recurrence (including locoregional or distant recurrence as detected clinically or radiologically), death from any cause, or the end of follow-up, whichever occurred first. Follow-up was conducted through outpatient visits, phone calls, and electronic medical records. The median follow-up time for survivors was 52.5 months (interquartile range: 33.6–71.4 months), with 85.0% of patients having at least 5 years of follow-up. The follow-up cutoff date was 31 May 2025 Patients with incomplete follow-up data were excluded from the analysis.

### 2.3 Construction and validation of the nomogram

Univariate and multivariate Cox regression analyses were performed to assess the impact of variables on OS and DFS in the training, internal validation, and external validation cohorts. The hazard ratio (HR) was calculated for each variable to identify independent risk factors. Variables with *p* < 0.05 in the univariate model were included in the multivariate Cox regression analysis. Nomograms were constructed based on the results of the multivariate Cox regression. To mitigate the risk of overfitting, we employed 10-fold cross-validation with 10 iterations in the training cohort. This method ensured that the model was evaluated across different subsets of the data, enhancing its generalization ability. The overall points for each patient in the training, internal validation, and external validation cohorts were calculated using the established nomogram, and a Cox regression analysis was then performed using the total points as a parameter. Additionally, internal and external cohort validation was conducted to assess the model’s stability and clinical applicability. These steps ensured the robustness and reliability of the nomogram across multiple datasets and cohorts.

### 2.4 The calibration curve and area under the receiver operating characteristic curve

The calibration of the nomogram was assessed using the Hosmer-Lemeshow test, with results displayed in the form of calibration curves. The evaluation criteria (39, 40) are provided in Supplementary Table 2. The predictive accuracy of the nomogram over time was represented by 1-, 3-, and 5-year receiver operating characteristic (ROC) curves, and the discriminative ability to predict OS and DFS in OCRC was quantitatively expressed as the area under the curve (AUC).

### 2.5 The concordance index and decision curve analysis

The concordance index (C-index) was defined as the ratio of all patient pairs for which the predicted outcomes are consistent with the observed results. Decision curve analysis (DCA) is a recently proposed method for evaluating the clinical value of a risk-prediction model. In this study, DCA was used to compare the clinical outcomes of the predictive nomograms.

### 2.6 Statistical analysis

Categorical variables were presented as counts and percentages, with comparisons made using the chi-square test or Fisher’s exact test. Survival differences were statistically analyzed using the log-rank test. Univariate and multivariate Cox regression analyses were performed to assess the impact of variables on OS and DFS. The HR was calculated for each variable. A stepwise backward elimination approach was applied in the multivariate Cox regression model, retaining factors with *p* < 0.05 from the univariate analysis. Statistical analyses were conducted using Statistical Package for the Social Sciences (SPSS) version 22.0 (IBM Corp., Armonk, NY, United States) and R version 4.0.2 (R Foundation for Statistical Computing, Vienna, Austria). The following R packages were used: “rms,” “survival,” “survminer,” “regplot,” “ggDCA,” “timeROC,” and “survcomp.” All statistical tests were two-sided, and statistical significance was set at *p* < 0.05.

## 3 Results

### 3.1 Prognosis and clinicopathological characteristics of OCRC patients

**FIGURE 2:**
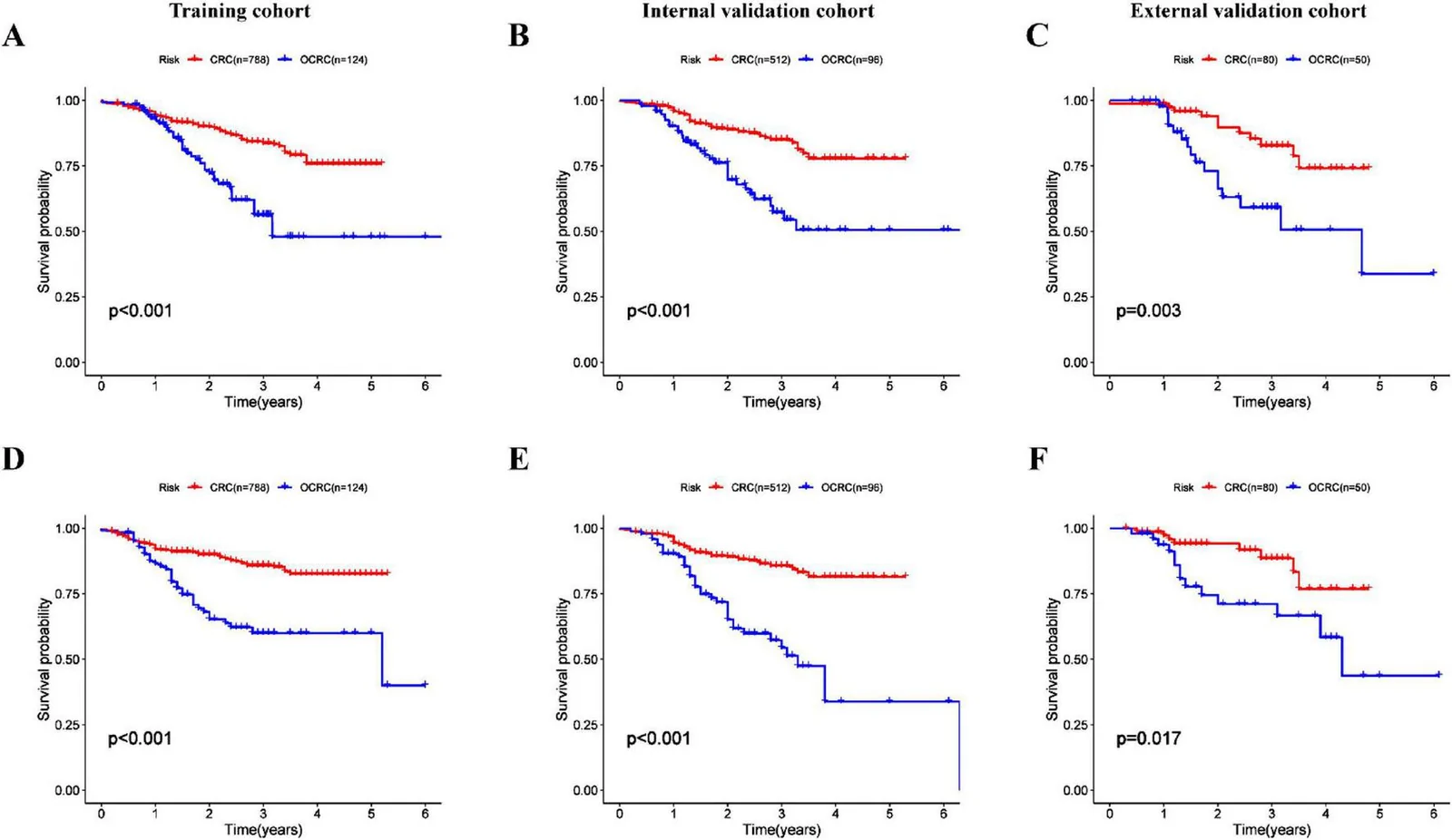
Comparison of overall survival (OS) and disease-free survival (DFS) between CRC and OCRC patients. OS differences in the training **(A)**, internal validation **(B)**, and external validation **(C)** cohorts; DFS differences in the training **(D)**, internal validation **(E)**, and external validation **(F)** cohorts.

Kaplan-Meier survival analysis demonstrated that OCRC patients had significantly lower overall survival (OS) (Figures 2A–C) and disease-free survival (DFS) (Figures 2D–F) compared to non-obstructive CRC patients across all cohorts (training, internal validation, and external validation). Specifically, for OS, the 5-year OS rate in the training cohort was 75.8% for CRC patients, compared to just 47.9% for OCRC patients (*p* < 0.001). In the internal validation cohort, CRC patients had a 5-year OS rate of 77.9%, while OCRC patients had 50.6% (*p* < 0.001). Similarly, in the external validation cohort, the 5-year OS rate for CRC patients was 72.9%, while for OCRC patients it was 43.8% (*p* < 0.01).

For DFS, in the training cohort, CRC patients had a 5-year DFS rate of 78.3%, while OCRC patients had 40.0% (*p* < 0.001). In the internal validation cohort, CRC patients had a 5-year DFS rate of 75.6%, compared to 33.9% in OCRC patients (*p* < 0.001). Lastly, in the external validation cohort, the 5-year DFS rate for CRC patients was 70.2%, while OCRC patients had 33.8% (*p* < 0.001).

In addition to survival differences, OCRC patients were found to have a more advanced clinical profile compared to non-OCRC patients. They presented with higher T and N stages, indicating more advanced local disease. Furthermore, lipid levels were significantly different, with OCRC patients exhibiting higher total cholesterol and lower HDL-C levels. OCRC patients also had a higher incidence of atherosclerosis-related indicators, such as AAS and CAS. These patients were generally older and had higher BMI compared to their non-OCRC counterparts. Interestingly, in both the training and internal validation cohorts, OCRC tumors were more often located in the colon and were, on average, smaller than those in non-OCRC patients, which may be related to the circumferential growth pattern characteristic of OCRC. These findings emphasize the distinct clinical and metabolic profile of OCRC patients and highlight the potential of lipid metabolism markers for prognostic evaluation (Table 1).

**TABLE 1:** Clinicopathological characteristics of patients in the training, internal validation and external validation cohorts [N (%)].

Characteristics	Category	Training cohort			Internal validation cohort			External validation cohort		
		CRC *N* = 788	OCRC *N* = 124	*P-*value	CRC *N* = 512	OCRC *N* = 96	*P-*value	CRC *N* = 80	OCRC *N* = 50	*P-*value
Age		–	–	<**0.001**	–	–	<**0.001**	–	–	**0.008**
	≤65	575 (73.0)	64 (51.6)	–	371 (72.55)	53 (55.2)	–	–	20 (40.0)	–
	>65	213 (27.0)	60 (48.4)	–	141 (27.5)	43 (44.8)		29 (36.2)	30 (60.0)	–
Gender		–	–	0.321	–	–	0.113	–	–	0.725
	Male	465 (59.0)	79 (63.7)	–	311 (60.7)	50 (52.1)	–	52 (65.0)	34 (68.0)	–
	Female	323(41.0)	45(36.3)	–	201 (39.3)	46 (47.9)	–	28 (35.0)	16 (32.0)	–
BMI		–	–	<**0.001**	–	–	**0.004**	–	–	**0.016**
	Low	47 (6.0)	21 (16.9)	–	30 (5.9)	10 (10.4)	–	6 (7.5)	9 (18.0)	–
	Normal	555 (70.4)	70 (56.5)	–	359 (70.1)	51 (53.1)	–	58 (72.5)	24 (48.0)	–
	Over	186 (23.6)	33 (26.6)	–	123 (24.0)	35 (36.5)		16 (20.0)	17 (34.0)	–
Differentiation		–	–	0.801	–	–	0.175	–	–	0.467
	Poorly differentiated	17 3(22.0)	24 (19.4)	–	131 (25.6)	24 (25.0)	–	15 (18.8)	7 (14.0)	–
	Moderately differentiated	562 (71.3)	91 (73.4)	–	357 (69.7)	63 (65.6)	–	61 (76.2)	38 (76.0)	–
	Well-differentiated	53 (6.7)	9 (7.2)	–	24 (4.7)	9 (9.4)	–	4 (5.0)	5 (10.0)	–
TNM stage		–	–	<**0.001**	–	–	<**0.001**	–	–	**0.007**
	Stage I	148 (18.8)	3 (2.4)	–	83 (16.2)	0 (0.0)	–	10 (12.5)	0 (0.0)	–
	Stage II	277 (35.1)	39 (31.5)	–	193 (37.7)	37 (38.5)	–	32 (40.0)	18 (36.0)	–
	Stage III	290 (36.8)	66 (53.2)	–	187 (36.5)	49 (51.0)	–	31 (38.8)	31 (62.0)	–
	Stage IV	73 (9.3)	16 (12.9)	–	49 (9.6)	10 (10.4)	–	7 (8.7)	1 (2.0)	–
T stage		–	–	<**0.001**	–	–	0.161	–	–	0.857
	T1	61 (7.7)	3 (2.4)	–	31 (6.1)	6 (6.3)	–	3 (3.8)	1 (2.0)	–
	T2	124 (15.7)	19 (15.3)	–	77 (15.0)	11 (11.5)	–	12 (15.0)	7 (14.0)	–
	T3	465 (59.0)	54 (43.6)	–	310 (60.6)	52 (54.2)	–	50 (62.5)	30 (60.0)	–
	T4	138 (17.5)	48 (38.7)	–	94 (18.4)	27 (28.1)	–	15 (18.8)	12 (24.0)	–
N stage		–	–	**0.002**	–	–	**0.006**	–	–	**0.045**
	N0	455 (57.7)	56 (45.2)	–	297 (58.0)	41 (42.7)	–	48 (60.0)	19 (38.0)	–
	N1	200 (25.4)	50 (40.3)	–	125 (24.4)	38 (39.6)	–	22 (27.5)	23 (46.0)	–
	N2	133 (16.9)	18 (14.5)	–	90 (17.6)	17 (17.7)	–	10 (12.5)	8 (16.0)	–
M stage		–	–	0.170	–	–	0.797	–	–	**0.047**
	M0	717(91.0)	108 (87.1)	–	463 (90.4)	86 (89.6)	–	74 (92.5)	50 (100.0)	–
	M1	71 (9.0)	16 (12.9)	–	49 (9.6)	10 (10.4)	–	6 (7.5)	0 (0.0)	–
Tumor location		–	–	<**0.001**	–	–	<**0.001**	–	–	0.818
	Left colon	176 (22.3)	40 (32.3)	–	107 (20.9)	35 (36.5)	–	27 (33.8)	17 (34.0)	–
	Right colon	161 (20.4)	57 (46.0)	–	125 (24.4)	35 (36.5)	–	22 (27.5)	16 (32.0)	–
	Rectum	451 (57.2)	27 (21.8)	–	280 (54.7)	26 (27.0)	–	31 (38.8)	17 (34.0)	–
Size, mm		–	–	**0.024**	–	–	**0.009**	–	–	0.824
	≤4	359 (45.6)	70 (56.5)	–	230 (44.9)	57 (59.4)	–	40 (50.0)	24 (48.0)	–
	>4	429 (54.4)	54 (43.5)	–	282 (55.1)	39 (40.6)	–	40 (50.0)	26 (52.0)	–
CRM		–	–	0.592	–	–	0.797	–	–	0.427
	No	777 (98.6)	123 (99.2)	–	505 (98.6)	95 (99.0)	–	79 (98.8)	50 (100.0)	–
	Yes	11 (1.4)	1 (0.8)	–	7 (1.4)	1 (1.0)	–	1 (1.2)	0 (0.0)	–
VI		–	–	**0.011**	–	–	0.181	–	–	**0.045**
	No	648 (82.2)	90 (72.6)	–	424 (82.8)	74 (77.1)	–	69 (86.3)	36 (72.0)	–
	Yes	140 (17.8)	34 (27.4)	–	88 (17.2)	22 (22.9)	–	11 (13.7)	14 (28.0)	–
PNI nerve		–	–	<**0.001**	–	–	**0.004**	–	–	**0.022**
	No	629 (79.8)	74 (59.7)	–	405 (79.1)	63 (65.6)	–	68 (85.0)	34 (68.0)	–
	Yes	159 (20.2)	50 (40.3)	–	107 (20.9)	33 (34.4)	–	12 (15.0)	16 (32.0)	–
TG^2^		–	–	0.279	–	–	**0.031**	–	–	**0.002**
	Normal	683 (86.7)	103 (83.1)	–	435 (85.0)	73 (76.0)	–	74 (92.5)	36 (72.0)	–
	High	105 (13.3)	21 (16.9)	–	77 (15.0)	23 (24.0)	–	6 (7.5)	14 (28.0)	–
CHOL		–	–	**0.002**	–	–	**0.019**	–	–	**0.003**
	Normal	670 (85.0)	92 (74.2)	–	434 (84.8)	72 (75.0)	–	71 (88.8)	34 (68.0)	–
	High	118 (15.0)	32 (25.8)	–	78 (15.2)	24 (25.0)	–	9 (11.2)	16 (32.0)	–
HDL-C		–	–	<**0.001**	–	–	<**0.001**	–	–	**0.001**
	Low	395 (50.1)	85 (68.6)	–	235 (45.9)	72 (75.0)	–	43 (53.7)	38 (76.0)	–
	Normal	202 (25.6)	27 (21.8)	–	152 (29.7)	12 (12.5)	–	19 (23.8)	12 (24.0)	–
	High	191 (24.2)	12 (9.7)	–	125 (24.4)	12 (12.5)	–	18 (22.5)	0 (0.0)	–
LDL-C		–	–	0.087	–	–	0.874	–	–	**0.034**
	Low	465 (59.0)	70 (56.4)	–	320 (62.5)	60 (62.5)	–	58 (72.5)	25 (50.0)	–
	Normal	145 (18.4)	16 (12.9)	–	89 (17.4)	15 (15.6)	–	7 (8.7)	8 (16.0)	–
	High	178 (22.6)	38 (30.6)	–	103 (20.1)	21 (21.9)	–	15 (18.7)	17 (34.0)	–
AC or AAS		–	–	<**0.001**	–	–	<**0.001**	–	–	<**0.001**
	AC	686 (87.1)	45 (36.3)	–	461 (90.0)	54 (56.2)	–	72 (90.0)	22 (44.0)	–
	AAS	102 (12.9)	79 (63.7)	–	51 (10.0)	42 (43.8)	–	8 (10.0)	28 (56.0)	–
CAC or CAS		–	–	**0.004**	–	–	<**0.001**	–	–	<**0.001**
	CAC	653 (82.9)	89 (71.8)	–	439 (85.7)	51 (53.1)	–	70 (87.5)	18 (36.0)	–
	CAS	135 (17.1)	35 (28.2)	–	73 (14.3)	45 (46.9)	–	10 (12.5)	32 (64.0)	–

CRC: non-obstructive colorectal cancer (no obstruction); OCRC: obstructive colorectal cancer (with obstruction). BMI, body mass index (≥18.5 and <24 as “Normal”); TNM, tumor-node-metastasis; CRM, circumferential resection margin; VI, vascular lymphatic invasion; PNI nerve, perineural invasion; TG: triglycerides (≥1.7 mmol/L as “High”); CHOL, total cholesterol (≥5.2 as “High”); HDL-C: high-density lipoprotein cholesterol (≥1.16 and <1.42 as “Normal”); LDL-C, low-density lipoprotein cholesterol (≥2.7 and <3.1 as “Normal”); AC, abdominal aortic calcification; AAS, abdominal aortic atherosclerosis; CAC, coronary artery calcification; CAS, coronary artery atherosclerosis. Bold values indicate *p* < 0.05.

### 3.2 Independent prognostic factors for OCRC patients

**FIGURE 3:**
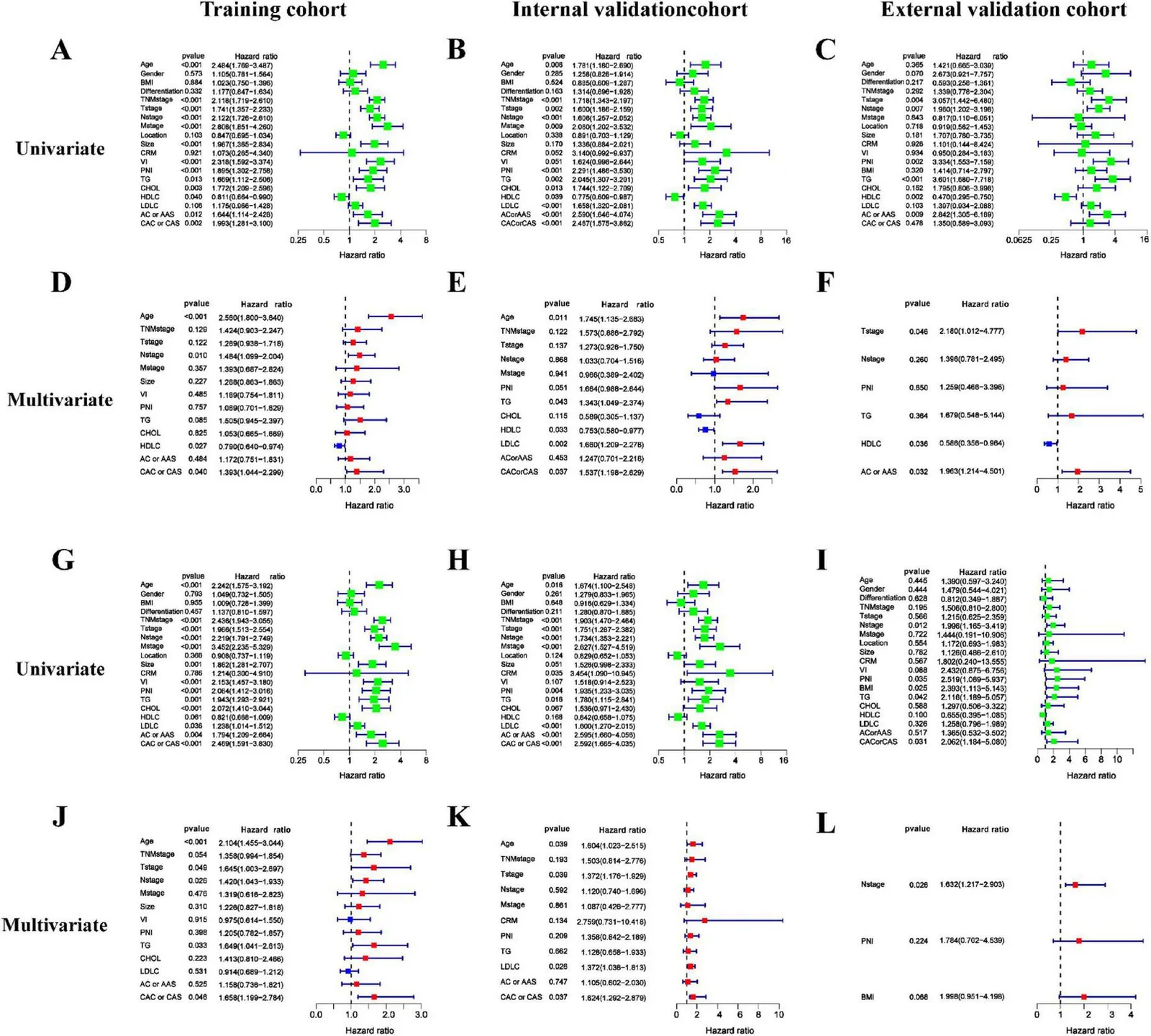
Univariate and multivariate Cox regression analysis for overall survival (OS) and disease-free survival (DFS) in the training, internal validation, and external validation cohorts. OS analysis **(A–F)**; DFS analysis **(G–L)**.

In the training cohort, univariate Cox regression analysis identified age, TNM stage, T stage, N stage, M stage, tumor size, VI, PNI nerve, TG, CHOL, HDL-C, AAS and CAS as significant factors associated with OS in OCRC patients (Figure 3A). Multivariate analysis revealed that age, N stage, and CAS were independent risk factors for OS, while HDL-C was an independent protective factor (HR = 0.790) (Figure 3D).

In the internal validation cohort, univariate analysis showed that age, TNM stage, T stage, N stage, M stage, PNI nerve, TG, CHOL, HDL-C, LDL-C, AAS and CAS were significantly associated with OS (Figure 3B). Multivariate analysis indicated that age, TG, LDL-C and CAS were independent adverse prognostic factors for OS, while HDL-C remained an independent protective factor (HR = 0.753) (Figure 3E).

In the external validation cohort, univariate analysis revealed that T stage, N stage, PNI nerve, TG, HDL-C and AAS were significantly associated with OS (Figure 3C). Multivariate analysis confirmed that T stage and AAS were independent risk factors for OS, whereas HDL-C was an independent protective factor (HR = 0.586) (Figure 3F).

For DFS, in the training cohort, multivariate Cox regression analyses identified age, T stage, N stage, TG, and CAS as independent adverse prognostic factors (Figure 3J). In the internal validation cohort, age, T stage, LDL-C and CAS were independent risk factors for DFS (Figure 3K). In the external validation cohort, N stage was an independent risk factor for DFS (Figure 3L).

### 3.3 Construction of the nomogram

**FIGURE 4:**
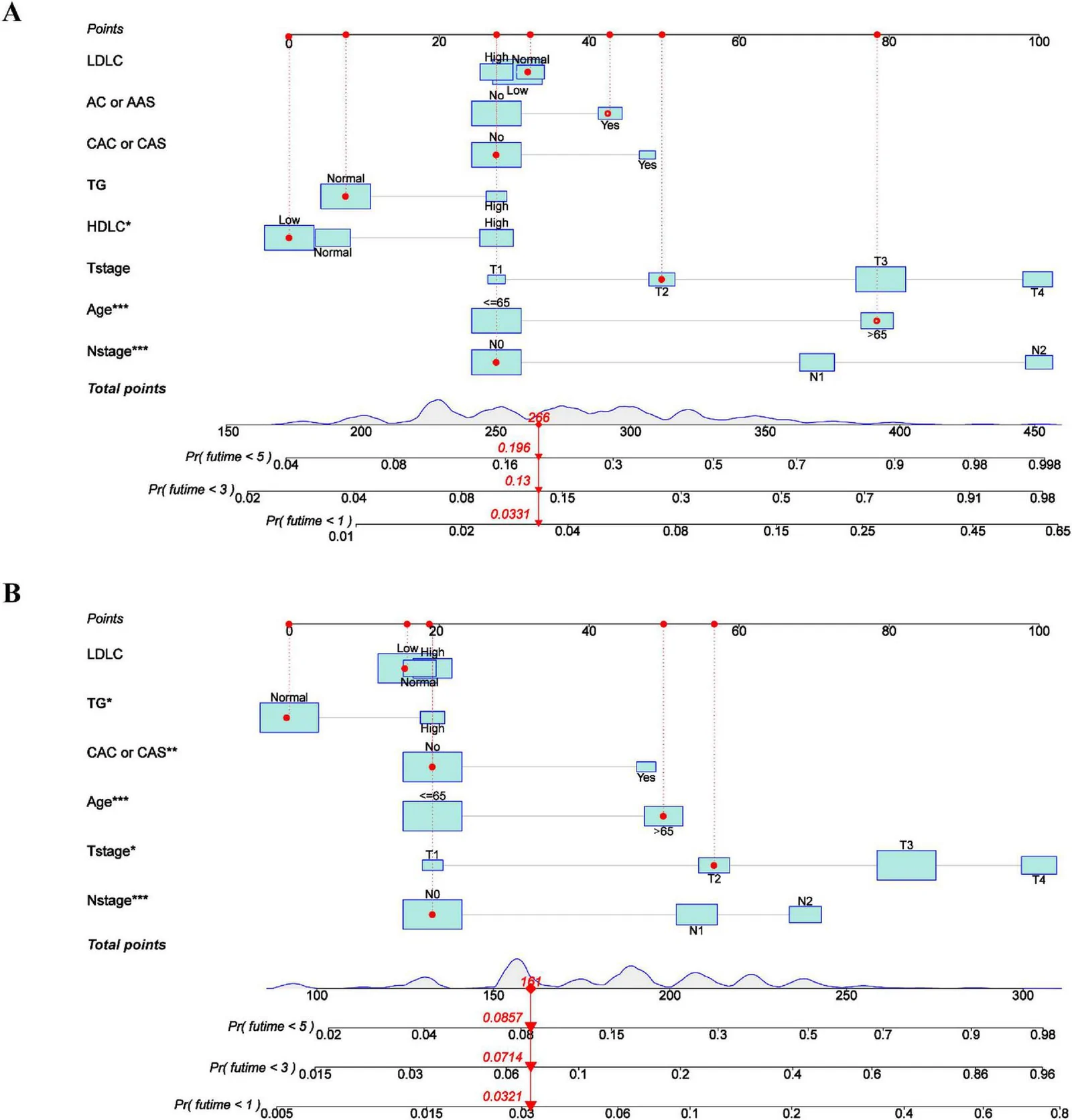
Nomograms for predicting outcomes in OCRC patients: **(A)** OS nomogram; **(B)** DFS nomogram, developed based on multivariate Cox regression analysis of the cohorts. *p < 0.05, **p < 0.01, ***p < 0.001.

Based on the results of multivariate Cox regression analysis, we constructed nomogram models for OS and DFS in patients with OCRC. The OS nomogram includes eight independent prognostic factors: LDL-C, TG, HDL-C, T stage, N stage, age, AAS and CAS (Figure 4A). The DFS nomogram incorporated LDL-C, TG, N stage, T stage, age, and CAS (Figure 4B).

In both nomograms, each variable was assigned a corresponding risk score, obtained from the scoring scale at the top of the chart. The total risk score was calculated by summing the individual risk scores, which was then used to estimate the patient’s overall risk, plotted on the total score axis at the bottom of the chart. Using this total score, the probabilities of OS and DFS at 1, 3, and 5 years can be predicted. The proportional hazards assumption of the Cox regression model was assessed using Schoenfeld residuals. The assumption was evaluated for each included lipid and clinicopathological variable, as well as for the global model. A non-significant Schoenfeld residual test was considered to indicate no evidence of violation of the proportional hazards assumption (OS, χ^2^ = 19.9, df = 13, *P* = 0.099; DFS, χ^2^ = 23.2, df = 16, *P* = 0.1056). For detailed information on the hazard ratios and their corresponding confidence intervals for each predictor, refer to Figure 3, which presents the results of the univariate and multivariate Cox regression analyses.

### 3.4 Comparison of the nomogram with individual prognostic indicators

**FIGURE 5:**
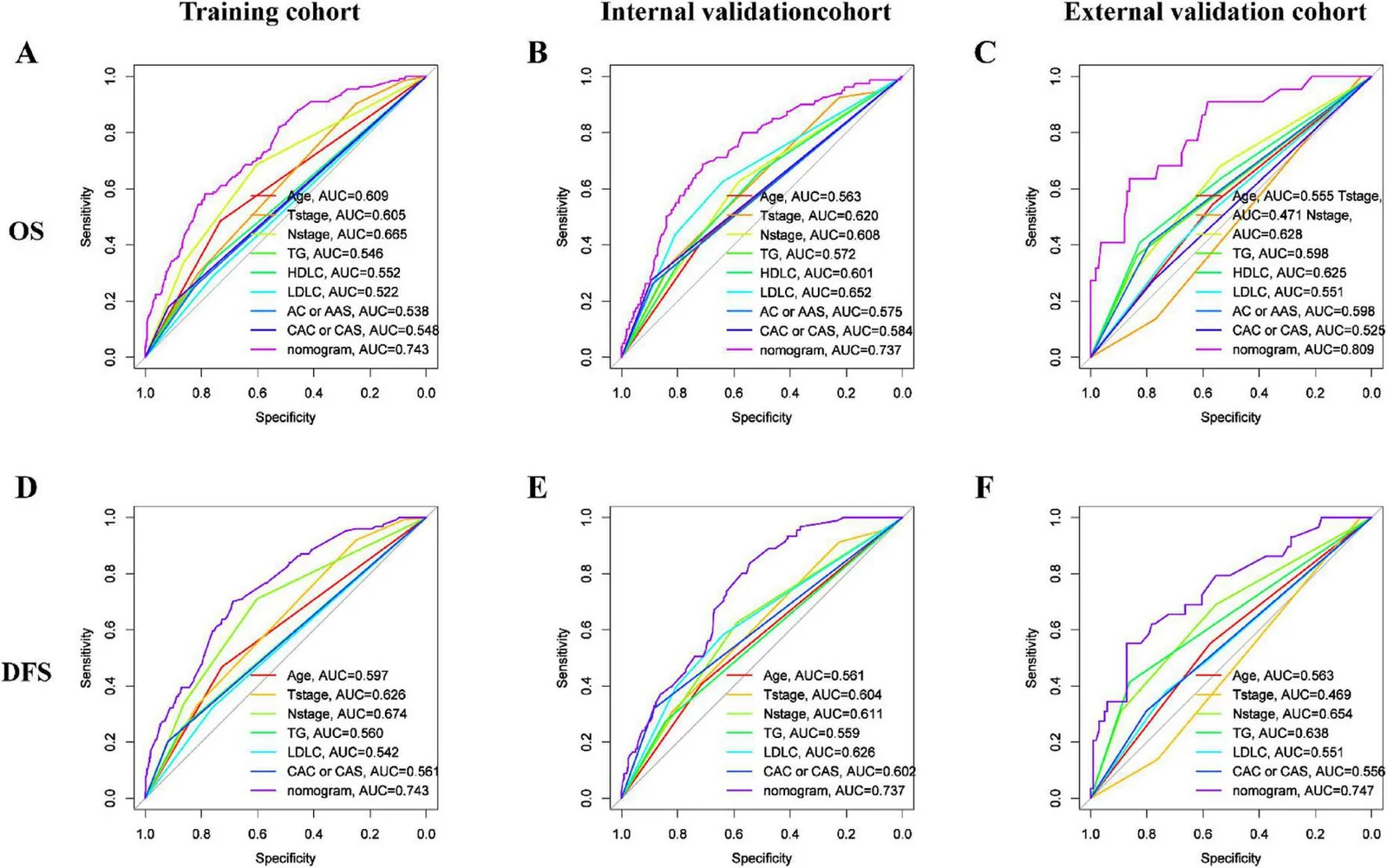
Receiver operating characteristic (ROC) curves comparing the performance of the nomogram and individual clinical features in predicting OS and DFS. OS for the training **(A)**, internal validation **(B)**, and external validation **(C)** cohorts; DFS for the training **(D)**, internal validation **(E)**, and external validation **(F)** cohorts.

To evaluate the predictive performance of the nomograms, we conducted receiver operating characteristic (ROC) curve analysis in the training, internal validation, and external validation cohorts. We compared the predictive ability of the nomogram models for each independent clinical and pathological prognostic factor. In the training cohort, the integrated AUC values for the OS and DFS nomograms across the entire follow-up period were 0.743 and 0.743, respectively, significantly surpassing the AUCs of each individual prognostic factor (Figures 5A,D). In the internal validation cohort, the nomogram outperformed each independent variable, with AUCs of 0.737 and 0.737 for OS and DFS, respectively (Figures 5B,E). In the external validation cohort, the AUC of the nomogram was 0.809 and 0.747 for OS and DFS, showing the best predictive performance across all comparisons (Figures 5C,F). These findings consistently indicate that the nomogram model offers stable and superior predictive accuracy across all cohorts. Notably, the incorporation of lipid metabolism indicators substantially enhanced the models’ prognostic capability.

### 3.5 Validation of the nomograms

To further validate the predictive performance of the nomograms, we conducted time-dependent ROC curve analysis to assess the predictive ability of OS and DFS at 1, 3, and 5 years in the training, internal validation, and external validation cohorts.

**FIGURE 6:**
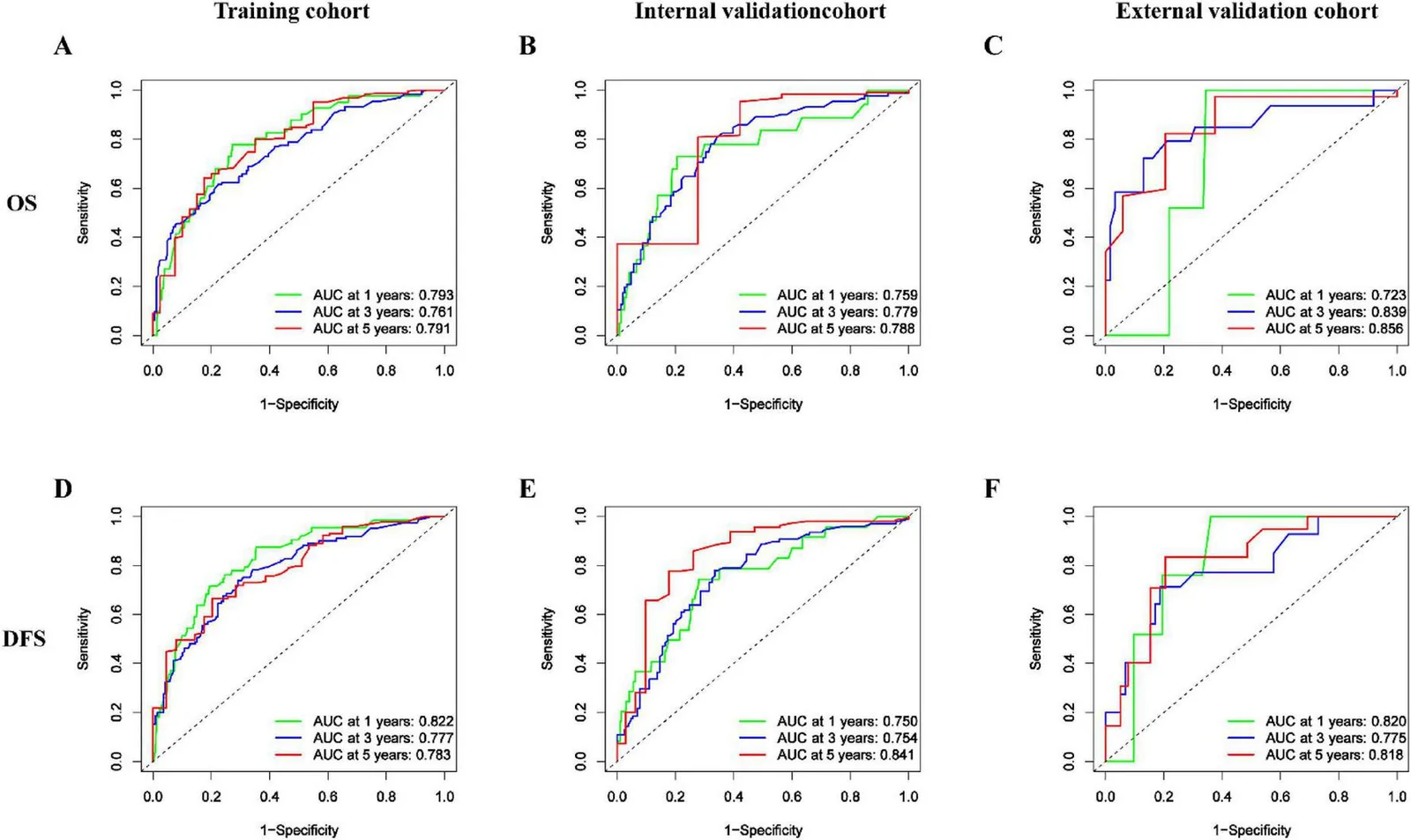
Time-dependent receiver operating characteristic (ROC) curves for the nomogram predicting OS and DFS: AUC values for 1-, 3-, and 5-year OS predictions in the training **(A)**, internal validation **(B)**, and external validation **(C)** cohorts; AUC values for 1-, 3-, and 5-year DFS predictions in the training **(D)**, internal validation **(E)**, and external validation **(F)** cohorts.

In the training cohort, the AUCs for the OS nomogram at 1, 3, and 5 years were 0.793, 0.761, and 0.791, indicating robust predictive performance. Similarly, the DFS nomogram performed well, with AUCs of 0.822, 0.777, and 0.783 at 1, 3, and 5 years, respectively. These results suggest strong discriminatory ability within the training cohort (Figures 6A,D).

In the internal validation cohort, the AUCs for the OS nomogram at 1, 3, and 5 years were 0.759, 0.779, and 0.788, and for the DFS nomogram, 0.750, 0.754, and 0.841(Figures 6B,E). Results from the external validation cohort further supported the models’ robustness, with AUCs for the OS nomogram of 0.723, 0.839, and 0.856 at 1, 3, and 5 years. The DFS nomogram showed AUCs of 0.820, 0.775, and 0.818 (Figures 6C,F).

In conclusion, the nomograms demonstrated consistent and stable predictive performance across multiple independent cohorts, confirming its excellent generalizability and potential for clinical application.

### 3.6 Concordance index and calibration

**FIGURE 7:**
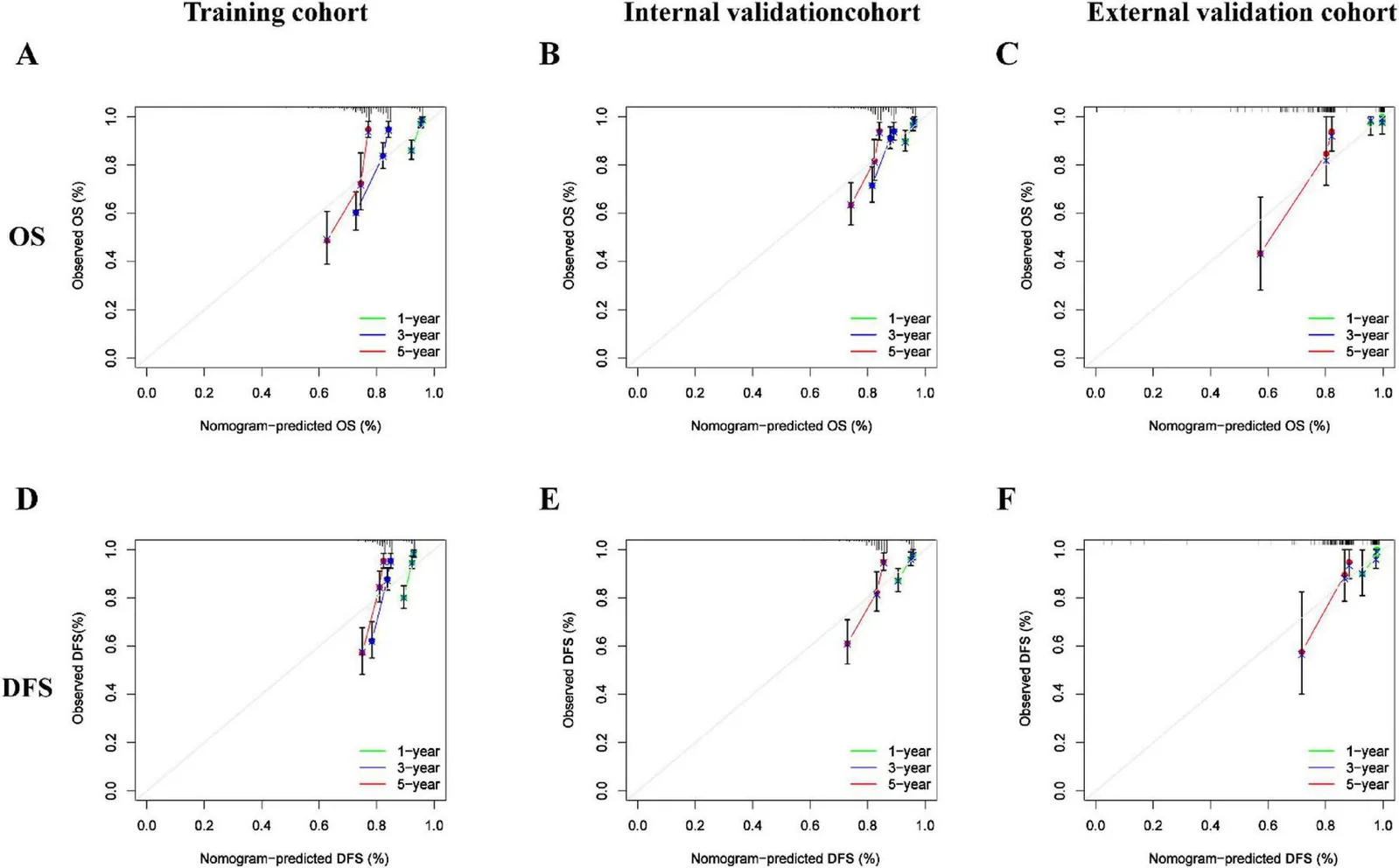
Calibration curves for the nomogram predicting 1-, 3-, and 5-year OS **(A–C)** and DFS **(D–F)** rates in the training, internal validation, and external validation cohorts.

Concordance index (C-index) analysis reveals that both the OS and DFS nomograms exhibited strong discriminative ability across the training, internal validation, and external validation cohorts. Specifically, the C-indexes for the OS nomogram were 0.751, 0.770, and 0.792 in the training, internal, and external validation cohorts, respectively. For the DFS nomogram, the C-indexes were 0.745, 0.741, and 0.753. To further assess the accuracy of the nomograms, calibration curves for OS and DFS at 1, 3, and 5 years were plotted (Figure 7). The results showed a close match between predicted and observed values across all cohorts. The corresponding intercept and slope values for each calibration curve are provided in Supplementary Table 3. The calibration curves for each time point were near the ideal reference line, reinforcing the predictive accuracy and stability of the model across different datasets.

### 3.7 Decision curve analysis

**FIGURE 8:**
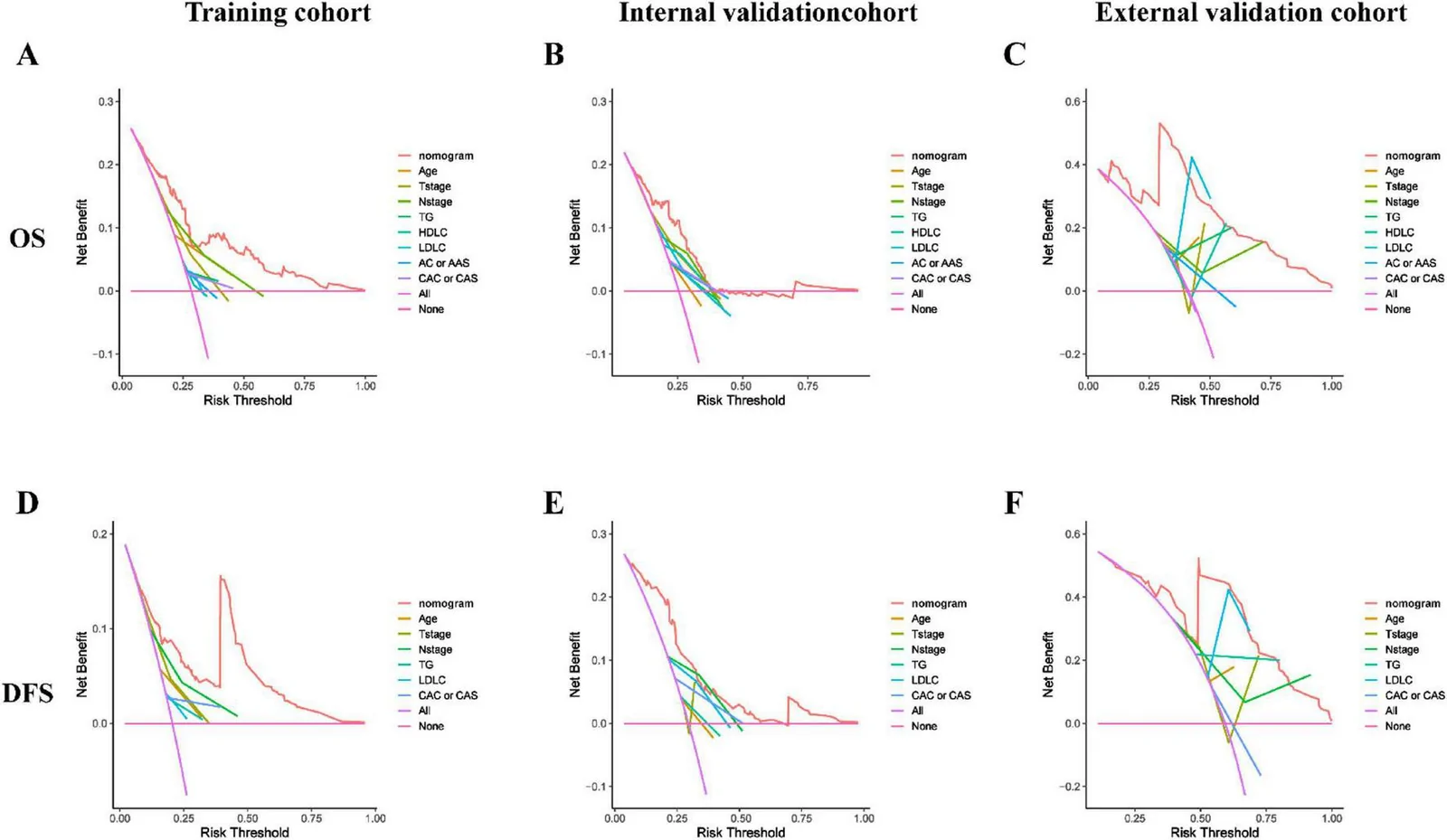
Decision curve analysis comparing the net benefit of the nomogram and individual clinical pathological factors in predicting OS **(A–C)** and DFS **(D–F)** in OCRC patients.

To evaluate the clinical applicability of the nomograms, we performed decision curve analysis (DCA) to compare the net benefit of the model with the traditional risk factors, such as the TNM staging system. The results indicated that across all risk threshold ranges, the net benefit of the OS and DFS nomograms was substantially higher than that of the TNM staging system, further supporting their clinical utility. This finding further suggests that the nomograms offer greater clinical applicability, particularly in personalized prognosis assessment and treatment decision-making for OCRC patients (Figure 8). Figure 8 shows the DCA curves (Figures 8A–C for OS and Figures 8D–F for DFS) demonstrating a higher net benefit for the nomogram (solid line) compared to TNM stage (dashed line) across thresholds. The DCA results suggest that, by using the nomograms, clinicians could make more informed decisions, such as identifying high-risk patients who might benefit from adjuvant chemotherapy or more intensive follow-up, compared to using TNM staging alone. In other words, for patients with a higher predicted risk, the nomogram provides added clinical value by guiding treatment escalation, thus optimizing individual care plans.

## 4 Discussion

We successfully developed and externally validated nomograms for predicting OS and DFS in patients with OCRC by integrating clinical lipid profiles (TG, HDL-C, and LDL-C), atherosclerosis-related indicators (AAS and CAS), and conventional clinical and pathological features (T stage, N stage, and age). The model demonstrated strong discrimination, calibration, and clinical utility across the training, internal validation, and external validation cohorts, offering a practical tool for preoperative risk assessment and personalized postoperative management of OCRC patients. Notably, this is the first study to incorporate lipid indicators into a prognostic model for OCRC patients, integrating lipid profiles with clinical and pathological data, and demonstrating strong predictive performance. Additionally, the multi-center, multi-cohort design of the study, enhances the validity and generalizability of our findings.

Due to the unclear underlying mechanisms of OCRC, existing prognostic models primarily rely on non-specific clinical and pathological factors, such as tumor markers (CEA, CA19-9), nutritional and inflammatory indicators (such as the PNI, NLR and LMR), and conventional prognostic risk factors (tumor stage and histological type) (22–25, 41, 42). The findings of this study further validated the stable prognostic value of traditional factors, such as TNM stage and age, in OCRC patients. Notably, based on our previous research on lipid dysregulation within tumors and its association with CRC matrix remodeling and obstruction progression (34), this study is the first to incorporate lipid indicators into the prognostic model for OCRC patients, offering new insights into potential biomarkers for prognosis.

Through univariate and multivariate Cox regression analyses, we identified HDL-C as an independent protective factor for OS in OCRC patients, whereas LDL-C and TG were independent risk factors for both OS and DFS. Numerous studies have established the association between lipid levels and cancer incidence and mortality, with lipid indicators being considered potential biomarkers for prognostic assessment in malignant tumors (43). However, despite growing interest in the relationship between lipid metabolism and prognosis across various cancer types, the prognostic value of lipid indicators varies considerably across different tumor types and research cohorts (44). The association of low HDL-C, high LDL-C, and high TG with poor prognosis in OCRC may reflect underlying metabolic dysregulation. HDL-C is known for its anti-inflammatory properties and may indicate better metabolic health, while high LDL-C and TG levels are often associated with metabolic syndrome, a pro-tumorigenic condition that promotes cancer progression. While our study does not address the potential for therapeutic intervention, managing lipid profiles through dietary changes or statin use could be a promising area for future research. Such interventions may help modify lipid metabolism, potentially improving OCRC patient outcomes. Further clinical trials would be valuable to explore whether lipid levels are merely markers of aggressive disease or if they represent modifiable risk factors that can be targeted therapeutically.

Some studies have suggested that preoperative serum cholesterol level is linked to prognosis in CRC, though its independent prognostic value has not been established (45). Other research has indicated that elevated HDL-C level may serve as an independent prognostic factor for OS and DFS in CRC patients post-chemotherapy, while decreased HDL-C level correlate with poor prognosis in CRC (46, 47). Additionally, high TG level have been shown to be significantly associated with poor prognosis in CRC patients (48). However, some studies have argued that the predictive value of lipid parameters for CRC prognosis remains controversial. For example, Chen et al. (48), Ye et al. (49) found no significant effect of HDL-C, LDL-C, CHOL, or TG levels on CRC prognosis. These contrasting conclusions emphasize the limitations of existing CRC prognostic models, which often address the entire CRC population without accounting for biological differences and clinical characteristics in subpopulations, such as OCRC. Therefore, developing more precise and targeted prognostic models for heterogeneous CRC populations is a crucial direction for future research.

Interestingly, tumor location and size also differed between OCRC and non-OCRC cases. OCRC tumors were more frequently located in the colon rather than the rectum, and paradoxically, OCRC tumors were smaller on average than non-obstructive tumors in the training and internal validation cohorts. This finding is intriguing, as one might expect larger tumors to cause obstruction. A potential explanation for this paradox could be the circumferential growth pattern of OCRC tumors, which may lead to lumen blockage even when the tumor itself is relatively small. This type of growth pattern is more likely to obstruct the bowel due to its ability to encircle and compress the bowel wall, causing significant luminal narrowing and obstruction. These observations highlight the complexity of OCRC and suggest that factors beyond tumor size, such as tumor growth patterns, should be considered when assessing prognosis and planning treatment strategies for OCRC patients.

Although BMI has been a subject of ongoing debate regarding its relationship with CRC prognosis (often discussed as the “obesity paradox”) (50–52), we demonstrated that BMI was not an independent prognostic factor for either OS or DFS in OCRC patients. While some literatures report that a high BMI correlates with an increased risk of local recurrence (53, 54). Additionally, some research indicates that both high and low BMI may contribute to poorer survival outcomes (55, 56). These discrepancies may stem from various factors, such as population heterogeneity, tumor site differences, staging distribution, surgical procedures, postoperative management strategies, and BMI measurement methods. BMI lost its statistical significance in our final models after adjusting for clinical lipid profiles and specific atherosclerosis indicators (AAS/CAS). This lack of independence in OCRC implies that systemic lipid metabolism abnormalities and macro-vascular changes may carry a much higher prognostic weight than BMI alone in this specific obstructive malignant subtype. Therefore, body mass index should be interpreted with caution and treated as a co-variable rather than an standalone predictor in OCRC risk stratification.

Age was found to be a significant independent risk factor for OS and DFS in both the training and internal validation cohorts. Older patients showed poorer survival outcomes, which is consistent with existing studies in various cancers that suggest aging is associated with worse prognosis due to factors like reduced immune function, increased comorbidity burden, and higher treatment-related complications (57). Clinically, this implies that older OCRC patients might require closer monitoring and potentially more aggressive interventions. However, further research is needed to explore the specific impact of age on OCRC prognosis.

The nomogram model constructed based on the identified independent risk factors demonstrates significant clinical translation potential. For example, in newly diagnosed OCRC patients, our nomogram can be used preoperatively to calculate their 1-, 3-, and 5-year overall survival (OS) and disease-free survival (DFS) probabilities. For high-risk patients, such as those who are older, have elevated triglyceride levels, low high-density lipoprotein (HDL), and more advanced tumor stages, they may be considered candidates for more aggressive adjuvant therapies or closer surveillance. Conversely, low-risk patients, such as those with younger age, lower triglyceride levels, and higher HDL, may avoid overly aggressive treatments. This personalized risk stratification helps tailor treatment intensity to the specific needs of the patient, ultimately optimizing outcomes while minimizing unnecessary interventions. By providing more accurate survival probability predictions, the nomogram can assist clinicians in making informed decisions regarding the timing and intensity of adjuvant therapies and post-treatment follow-up schedules.

Although this study provided new prognostic models and validated their clinical effectiveness, it had several limitations. First, the external validation cohort had a relatively small sample size and was derived from a single center, which may limit the models’ external applicability. Second, as this study was retrospective, prospective multi-center studies are necessary to further validate the models’ robustness and generalizability, especially across diverse racial and geographic populations. If validated, the integration of these models into clinical practice—such as through an online calculator or other digital platforms—could provide immediate benefits in OCRC management. This could enable clinicians to make more informed, personalized treatment decisions, ultimately improving patient outcomes and optimizing treatment strategies. Third, this study only included OCRC patients who underwent radical surgery, and did not cover those who were unresectable or only managed palliatively. Therefore, the nomogram developed in this study is applicable only to surgical candidates and does not apply to non-operative cases. Additionally, this study did not sufficiently account for potential confounders and missing variables, such as perioperative treatments (e.g., chemotherapy, stenting strategies, and the choice between emergency versus elective surgery) and molecular markers (e.g., microsatellite instability and RAS/RAF mutations). These factors may also have a significant impact on the prognosis of OCRC patients. From a metabolic standpoint, immediate ER under acute mechanical crisis triggers an intense systemic stress response and inflammatory cascades, which can heavily accelerate lipid mobilization (such as stress-induced lipolysis) and acutely distort circulating TG or cholesterol fractions. Conversely, a planned elective surgery after successful BTS decompression allows a physiological stabilization window, potentially resetting the patient’s baseline lipid profile closer to their true metabolic homeostasis, future prospective multi-center trials must systematically archive the exact timing and modalities of surgical interventions to clarify whether the nomograms’ predictive performance exhibits subtle performance shifts across distinct acute management windows. Future studies should aim to incorporate these factors to enhance the models’ clinical applicability and ensure more comprehensive risk stratification. We also did not include some commonly used prognostic markers in CRC, such as CEA and inflammatory indices (e.g., NLR). While these markers are often used in CRC prognostication, they were not included in this study because prior comparisons between CRC and OCRC patients found no significant differences for markers like CEA. The primary aim of our study was to investigate the prognostic impact of lipid metabolism markers in OCRC patients specifically, based on the CRC background. However, in broader CRC populations or in studies not targeting a specific subset like OCRC, these classical markers should still be considered and analyzed for their potential role in prognosis. Moreover, the current model does not integrate imaging indicators or dynamic monitoring metrics. Future research should incorporate multi-omics data and artificial intelligence algorithms to further optimize the models’ performance.

## 5 Conclusion

In summary, this study constructed and validated nomogram models for postoperative prognostic assessment in patients with OCRC. As evidenced by the net benefit from DCA, these models demonstrate superior predictive performance compared to conventional staging alone, offering an accurate risk prediction tool for high-risk individuals. Additionally, the models highlight the potential link between lipid metabolism and OCRC prognosis, which could guide the development of metabolic intervention strategies. Prospective studies are needed to further refine these models and facilitate their adoption in clinical practice.

## Data Availability

The raw data supporting the conclusions of this article will be made available by the authors, without undue reservation.
