# Correction: Lipid profile-based prognostic nomograms for obstructive colorectal cancer: multi-cohort development and validation

**DOI:** 10.3389/fmed.2026.1951852

**Published:** 2026-08-11

**Authors:** Hanwenchen Wang, Falong Zou, Denglong Cheng, Jun Wang, Mian Chen, Junnan Gu, Fuwei Mao, Kun Dai, Ke Wu, Hongli Liu, Xiu Nie, Li Liu, Jianguo Shi, Shenghe Deng, Kailin Cai

**Affiliations:** 1 Department of Gastrointestinal Surgery, Union Hospital, Tongji Medical College, Huazhong University of Science and Technology, Wuhan, Hubei, China; 2 Department of Thoracic Surgery, Union Hospital, Tongji Medical College, Huazhong University of Science and Technology, Wuhan, Hubei, China; 3 School of Nursing, Wuhan University, Wuhan, China; 4 Cancer Center, Union Hospital, Tongji Medical College, Huazhong University of Science and Technology, Wuhan, Hubei, China; 5 Department of Pathology, Union Hospital, Tongji Medical College, Huazhong University of Science and Technology, Wuhan, Hubei, China; 6 Department of Epidemiology and Biostatistics, School of Public Health, Tongji Medical College, Huazhong University of Science and Technology, Wuhan, China; 7 Department of Gastrointestinal Surgery, The Central Hospital of Wuhan, Tongji Medical College, Huazhong University of Science and Technology, Wuhan, China; 8 Department of General Surgery, Union Hospital, Tongji Medical College, Huazhong University of Science and Technology, Wuhan, Hubei, China; 9 Center for Liver Transplantation, Union Hospital, Tongji Medical College, Huazhong University of Science and Technology, Wuhan, Hubei, China

**Keywords:** colorectal cancer, disease-free survival (DFS), lipid profile, nomogram, obstruction, OS

Authors Hanwenchen Wang, Falong Zou, and Denglong Cheng were erroneously omitted as equal contributing authors.

The original version of this article has been updated.

