# Supplementary material for: Lipid profile-based prognostic nomograms for obstructive colorectal cancer: multi-cohort development and validation
Source: Front Med (Lausanne). 2026 Jul 27;13:1910496. doi: 10.3389/fmed.2026.1910496 (PMC13454900; doi:10.3389/fmed.2026.1910496)
Supplement: Supplementary file 1 [file Table_1.docx]

**Supplementary table 1**

Stratification criteria of BMI and blood lipid indicators

|  | **BMI (kg/m^2^)** | | **TG (mmol/L)** | **CHOL (mmol/L)** | **HDLC (mmol/L)** | **LDLC (mmol/L)** |
| --- | --- | --- | --- | --- | --- | --- |
| **Low** | ＜18.5 | ＜1.7 | | ＜5.2 | ＜1.16 | ＜2.7 |
| **Normal** | ≥18.5 and ＜24 |  | |  | ≥1.16 and ＜1.42 | ≥2.7 and ＜3.1 |
| **High** | ≥24 | ≥1.7 | | ≥5.2 | ≥1.42 | ≥3.1 |

BMI: body mass index; TG: triglycerides; CHOL: total cholesterol; HDL-C: high-density lipoprotein cholesterol; LDL-C: low-density lipoprotein cholesterol
