# Supplementary material for: Lipid profile-based prognostic nomograms for obstructive colorectal cancer: multi-cohort development and validation
Source: Front Med (Lausanne). 2026 Jul 27;13:1910496. doi: 10.3389/fmed.2026.1910496 (PMC13454900; doi:10.3389/fmed.2026.1910496)
Supplement: Supplementary file 2 [file Table_2.docx]

**Supplementary table 2**

Calibration curve evaluation standard for model performance assessment based on intercept and slope.

| **Index** | **Value** | **Evaluation** | **Model Performance** |
| --- | --- | --- | --- |
| **Calibration Intercept** | 0 | Ideal | Perfect |
|  | -0.05 to 0.05 | Acceptable | Excellent |
|  | -0.1 to 0.05 or 0.05 to 0.1 | Cautionary | Good |
|  | ＜-0.1 or ＞0.1 | Problematic | Poor |
| **Calibration Slope** | 1 | Ideal | Perfect |
|  | 0.9 to 1.1 | Acceptable | Excellent |
|  | 0.8 to 0.9 or 1.1 to 1.2 | Cautionary | Good |
|  | ＜0.8 or ＞1.2 | Problematic | Poor |
