# Supplementary material for: Lipid profile-based prognostic nomograms for obstructive colorectal cancer: multi-cohort development and validation
Source: Front Med (Lausanne). 2026 Jul 27;13:1910496. doi: 10.3389/fmed.2026.1910496 (PMC13454900; doi:10.3389/fmed.2026.1910496)
Supplement: Supplementary file 3 [file Table_3.docx]

**Supplementary table 3**

Calibration Intercepts and Slopes for OS and DFS Nomograms

| **OS/DFS** | **Cohorts/Years** | **Calibration Intercept** | **Calibration Slope** |
| --- | --- | --- | --- |
| **OS** | Training-1 | -0.022 | 1.034 |
|  | Training-3 | -0.057 | 1.053 |
|  | Training-5 | -0.071 | 1.108 |
|  | Internal validation-1 | -0.019 | 1.029 |
|  | Internal validation-3 | -0.063 | 1.073 |
|  | Internal validation-5 | -0.085 | 1.097 |
|  | External validation-1 | 0.064 | 1.054 |
|  | External validation-3 | -0.068 | 1.078 |
|  | External validation-5 | -0.095 | 1.134 |
| **DFS** | Training-1 | -0.038 | 1.051 |
|  | Training-3 | -0.056 | 1.070 |
|  | Training-5 | -0.077 | 1.093 |
|  | Internal validation-1 | -0.013 | 1.020 |
|  | Internal validation-3 | -0.035 | 1.047 |
|  | Internal validation-5 | -0.047 | 1.062 |
|  | External validation-1 | -0.021 | 1.018 |
|  | External validation-3 | -0.050 | 1.061 |
|  | External validation-5 | -0.061 | 1.097 |

OS: Overall survival; DFS: Disease-free survival
